# Description and evaluation of a French grief workshop for children and adolescents bereaved of a sibling or parent

**DOI:** 10.1186/s12904-021-00861-9

**Published:** 2021-10-15

**Authors:** Ashley Ridley, Alexis Revet, Jean-Philippe Raynaud, Eric Bui, Agnès Suc

**Affiliations:** 1 Department of Paediatric Palliative Care, Toulouse University Hospital, Toulouse, France; 2 Department of Child and Adolescent Psychiatry, Toulouse University Hospital, Toulouse, France; 3 Centre for Epidemiology and Population Research, University of Toulouse, Inserm, UPS, Toulouse, France; 4 Department of Psychiatry, Massachusetts General Hospital, Boston, MA USA; 5 University of Caen Normandy & Caen University Hospital, Caen, France

**Keywords:** Bereavement, Child, Paediatrics, Surveys and questionnaires

## Abstract

**Background:**

Childhood bereavement is common, and is associated with elevated symptoms of grief with distress and impairment. However, few developmentally appropriate interventions to support grieving children are available to date. In Toulouse, France we developed an innovative four-session group intervention to support grieving families and evaluated its feasibility and acceptability.

**Methods:**

The workshop consists of four sessions over 4 months, open to children bereaved of a sibling or parent, and co-facilitated by two mental health professionals. After an intake assessment, children were placed into closed groups according to age and relation to the deceased. The session content was balanced between creative activities and grief-related discussions. Overall satisfaction was evaluated in March-April of 2020 by an 8-question online survey of children and parents having participated between 2011 and 2019. Freeform commentaries were analysed using the thematic synthesis process.

**Results:**

Of the 230 emails sent in March 2020, 46 children and 81 parents agreed to participate (55% response rate). The families reported an overall high level of satisfaction regarding the intervention that was rated as good to excellent. A majority of respondents considered their participation in the workshop helpful and in accordance with their expectations. Most would recommend the workshop to a friend, and would participate again in the group if needed. The group intervention helped reduce social isolation, facilitated grief expression, and supported the creation of a sense of community among bereaved families.

**Conclusions:**

Encouraging community and mutual support among grieving families is fundamental in bereavement care. Our four-session workshop held over 4 months and led by mental health professionals aimed to help reduce social isolation and foster coping skills through artistic creation and group discussion. Our results highlight the potential need for family bereavement support over a longer period and a provision of a variety of services. Our intervention model is feasible for families, and further studies examining its efficacy are warranted.

## Introduction

Childhood bereavement is prevalent and can be associated with elevated symptoms of grief with distress and impairment. A British study estimated that 3.5% of children in the UK, or one child per school classroom, is coping with grief following the death of an immediate family member (parent or sibling) [1]. Despite being a universal experience, bereavement can negatively affect both short- and long-term psychosomatic and socioeconomic outcomes on an individual level [2]. Most commonly encountered long-term manifestations and challenges are as follows: difficulty falling asleep [3–5], anxiety and depression [3, 4, 6–8], internalizing and externalizing behavior disorders [9, 10], low self-esteem [3, 11], social withdrawal [5, 9], increased absence from school [12, 13], poor academic performance [14], illicit substance use [6, 15, 16] and attempted suicide [6, 17]. Furthermore, unsatisfactory social support, namely a lack of communication with family and peers regarding the death, can be a leading risk factor for increased anxiety and prolonged grief [7, 18–21]. On the other hand, some grief counselors argue that positive outcomes can emerge from bereavement such as posttraumatic growth, increased resilience, pro-social behavior and altruism [22].

An accurate conception of death is generally acquired between ages 9 and 11 [9, 23]. During their cognitive development, children progressively ascertain five concepts to comprehend death: irreversibility (death is permanent), personal mortality (death applies to oneself), universality (death is inevitable), non-functionality (with death all life functions cease), and causality (realistically understanding death’s causes) [9, 23]. At each milestone, grief is revisited and redefined. A child’s coping mechanisms gradually become more operational and effective throughout life [9, 23]. Inevitably, a portion of the grieving process is delayed and resolved at a later point in life [2, 24]. However, complicated grief is evoked when there are persistent intense symptoms of acute grief and/or thoughts, feelings or behaviors reflecting excessive or distracting concerns about the circumstances or consequences of the death [25]. Being particularly attentive to risk factors for complicated grief preceding the death (close relation to the deceased, history of difficult family relationships, prior personal mental health history), circumstances surrounding (violent and/or unexpected circumstances, absent or forced participation in funeral rites) and/or following the death itself (changes in daily life patterns, adverse reactions of family and peers) is important for correct onward referral to professional care where needed [5].

Although the majority of bereaved children do not show any signs of serious disturbance, developmentally appropriate bereavement care for children is helpful. This may include external professional help and supportive peer/family groups to help navigate the grief experience [21, 26]. Bereavement interventions can take on various forms, such as group sessions, weekend camps, family therapy and individual therapy [27]. Preliminary qualitative and quantitative results of group intervention evaluations (multiple sessions or bereavement camp) show a positive effect for families. Primary benefits include creating a sense of community among grieving families, and facilitating dialogue and mutual encouragement [27]. A recent review of the literature of bereavement care interventions for siblings under the age of 18 showed that the group intervention “*Histoire d’en Parler*” (“*Let’s talk about it”*) is the only French intervention described in scientific literature [27]. The conception of the workshops stemmed from a growing concern of lack of local support for bereaved siblings. Although children might not present with complicated grief nor need professional help, no other form of support was available other than individual psychological or psychiatric care. Furthermore, local non-profit organisations providing support for bereaved adults felt unable to extend their care to children and adolescents. The paediatric palliative care team and the child psychiatry team collaborated with an expert psycho-sociologist in grief care to create the bereavement workshop. Initially the workshops were open only to children bereaved of a sibling. However, as the demand increased additional groups for children bereaved of a parent were opened. Children bereaved by parental suicide were also gradually included in the workshops. The workshop was comprised of four group sessions over 4 months and provided bereaved children the opportunity to share their experiences through art-based activities and mediated discussions [28]. The objective of our study was to evaluate overall feasibility and acceptability of the workshop.

## Materials and methods

We conducted a retrospective online satisfaction survey of families who participated in the “*Let’s talk about it*” bereavement workshop organized collaboratively by the pediatric palliative care and child psychiatry teams of a tertiary referral hospital in the south of France.

### Study methodology

A closed-ended online survey was emailed in March-April of 2020 to all children and parents who participated in the workshops between 2011 and 2019. Initial contact with potential participants was made by email to obtain informed consent, and a second reminder email was sent 2 weeks later. Participants were informed of the study’s investigators, purpose, methodology, duration, data storage and protection. No personal data was collected. Those who agreed to participate were then sent a link to the survey by email. All children (6-18 years of age) and their parents (or legal guardians) who completed the workshops between 2011 and 2019 were eligible for inclusion. Families with incorrect or out of date contact information, families not wishing to participate in the study, and those from whom we received no response were excluded.

Two surveys were prepared for participating family members—one for children and one for parents. The developmentally-appropriate children’s survey was launched with simple language and pictograms that could be completed autonomously from age six. Each survey comprised eight closed-ended questions to evaluate user satisfaction with the option of writing free-form commentaries at the end of the survey, if desired. Each question had a range of four answers rated on a four-point Likert scale. The clarity of the survey questions and the technical functionality of the online questionnaire were tested internally by the research team prior to fielding the survey.

The survey responses were analysed using the software SPHINX iQ2 version 7.4.3.0. This software was employed for creating the online survey, distributing the link via email, collecting data, and generating descriptive statistical analysis. Our results were reported according to the Checklist for Reporting Results of Internet E-Surveys (CHERRIES) [29]. A qualitative analysis of the free-form commentaries was conducted using the thematic synthesis process [30]. We first coded text “line-by-line” from the commentaries to create “free codes” (without hierarchal structure). These codes were then regrouped into related areas to construct “descriptive themes” close to the original text (for example: workshop length, group dynamic). From these themes emerged inferred “analytical themes”. These themes go beyond the original text to generate additional understandings and concepts regarding the bereavement intervention (for example: need for extended follow-up, reducing isolation) [30].

The study was approved by the local ethics committee (Institutional Clinical Research and Innovation Review Board (reference number RnIPH 2020-55)).

### Description of the workshop

The family and child first undergo a pre-intervention screening process by consultation with a pediatrician. The purpose of this intake assessment is to take medical history, to understand the circumstances of the death and the child’s understanding of grief, and to explain the intervention layout to prospective participants. If the child does not seem apt to share their grief in a group setting, they are referred onward for more appropriate care.

The workshop involves four evening sessions over a period of 4 months. Each session lasts approximately 90 min. The workshop is a closed group of six to eight participants divided into age groups (6-12 years and 13-18 years). They are co-facilitated by two trained staff members (child psychologists or child psychiatrists). Staff members receive an intensive one-day training course on childhood grief prior to leading workshop sessions. In addition, each member is trained in methodology on leading group discussion. All new staff members are paired with a more experienced staff member during their first year of group leadership for encouragement and support. Following each workshop session is a debriefing with the staff members involved, conducted by an external child psychiatrist with group leadership experience. All staff members meet twice annually to discuss and evaluate the workshop process and make any necessary changes.

The overall outline of each session is similar and is reproducible from one workshop to another. The sessions begin with a time for individual creativity, followed by a mediated grief-centered group discussion, and concluding with a collective painting project. The creative activities take place in an arts and crafts room, whereas the discussions take place in a more intimate and cozy environment. The arts and crafts activity goals are as follows: drawing a picture of their deceased loved one, coloring mandalas where the colors represent different emotions, cut-and-paste collage of their family and their future, and making clay figures of their loved ones or of memorable objects. Although the arts activities are planned, session discussion topics are not pre-determined but rather stem from the group sharing. This requires the staff to be able to adapt to each individual group’s needs, but equally allows the participants the opportunity and space to voice their concerns and questions. In our experience, each group is unique and different, but there are several recurrent themes- how to talk about grief in a social setting, school related issues, family-dynamic related issues, continuing bonds, fighting isolation and awkwardness especially during adolescence, survivor’s guilt, fear of losing another loved one, and existential questions about life and death. The intention of these discussions is to help children express and normalize their grief, to help reduce isolation and to facilitate their grieving process.

At the end of the four sessions, the family is invited to meet again with the pediatrician who completed their intake assessment. The therapist provides a behavioral summary report for each child, identifying concerns and progress during the workshops. If needed, the child will be referred onward for one-on-one professional psychological care.

During the workshops, an optional discussion group is offered to parents. This parallel group is led by volunteers from a bereavement support organization and takes place in a nearby separate location from the children’s workshops.

The goal of the workshop format is to help children identify and develop coping mechanisms that facilitate adjustment to a significant person’s death. This focus includes primarily social support and normalization, memory activities, and fostering resilience. Firstly, creating community among grieving children helps to destigmatize bereavement and reduce the feeling of isolation. Secondly, grief-centered commemorative activities help to create continuing bonds with the deceased. Studies indicate that talking openly, cherishing mementos and forming continuing bonds with the deceased can be associated with better adjustment to significant loss [31]. The artistic activities aim to help the child develop a coherent narrative of their loss and thus minimize maladaptive feelings and behaviors surrounding the death. Lastly, the group discussions and activities were designed to encourage children to share their grief experience, normalize their feelings and develop resilience and coping skills.

## Results

Of the 230 emails sent in March 2020, 46 children (aged 6 to 15 at time of participation) and 81 parents agreed to participate (55% response rate). Participation was declined by 48 people. For 55 people, the contact information was incorrect or out of date. The response rate of families with current contact information was 75%. The breakdown of responses to each question is detailed in Table 1 for the children and Table 2 for the parents.

**Table 1:** Children’s responses

**Questions**	**Responses**			
1. What do you think about the workshops?	Excellent (*n* = 16, 35.8%)	Good (*n* = 24, 52.2%)	Average (*n* = 6, 13.0%)	Poor (*n* = 0, 0%)
2. Did the workshops meet your expectations?	Yes, entirely (*n* = 11, 23.9%)	Yes, partially (*n* = 27, 58.7%)	No, not really (*n* = 8, 17.4%)	No, not at all (*n* = 0, 0%)
3. Did the workshops help you?	Yes, a lot (*n* = 20, 43.5%)	Yes, partially (*n* = 18, 39.1%)	Only a little bit (*n* = 6, 13.0%)	Not at all (*n* = 2, 4.3%)
4. Would you recommend the workshops to a friend?	Yes definitely (*n* = 25, 54.3%)	Yes, maybe (*n* = 16, 34.8%)	No I don’t think so (*n* = 5, 10.9%)	No, definitely not (*n* = 0, 0%)
5. Are you happy with the help you received?	Yes, very happy (*n* = 18, 39.1%)	Yes, mostly happy (*n* = 17, 37.0%)	Indifferent (*n* = 11, 23.9%)	Not at all happy (*n *= 0, 0%)
6. Did the workshops help you deal with your problems?	Yes, it helped a lot (*n* = 10, 21.7%)	Yes, it helped some (*n* = 25, 54.3%)	No, it didn’t help (*n* = 11, 23.9%)	No, it was even worse afterwards (*n *= 0, 0%)
7. Overall, are you happy to have participated in the workshops?	Yes, very happy (*n* = 21, 45.7%)	Yes, somewhat happy (*n* = 22, 47.8%)	No, not very happy (*n* = 3, 6.5%)	No, very upset (*n* = 0, 0%)
8. If you needed help in the future, would you chose to participate in the workshops again?	Yes, without hesitation (*n* = 17, 37.0%)	Yes, I think so (*n* = 10, 21.7%)	I don’t know (*n* = 17, 37.7%)	No, never (*n* = 2, 4.3%)

**Table 2:** Parents’ responses

**Questions**	**Responses**			
1. How would you rate the quality of the workshops?	Excellent (*n* = 45, 55.6%)	Good (*n* = 30, 37.0%)	Average (*n* = 5, 6.2%)	Poor (*n* = 1, 1.2%)
2. Did the workshops meet your expectations?	Yes, entirely (*n* = 39, 48.1%)	Yes, partially (*n* = 35, 43.2%)	No, not really (*n* = 6, 7.4%)	No, not at all (*n* = 1, 1.2%)
3. To what extent were has our program met your needs?	Almost all my needs were met (*n* = 20, 24.7%)	Most of my needs were met (*n* = 42, 51.9%)	Some or few of my needs were met (*n* = 17, 21.0%)	None of my needs were met (*n* = 2, 2.5%)
4. Would you recommend the workshops to a friend?	Yes definitely (*n* = 60, 74.1%)	Yes, probably (*n* = 16, 19.8%)	No I don’t think so (*n* = 4, 4.9%)	No, definitely not (*n* = 1, 1.2%)
5. Are you satisfied with the amount of help you received?	Yes, very satisfied (*n* = 38, 46.9%)	Yes, mostly satisfied (*n* = 32, 39.5%)	Indifferent or not really satisfied (*n* = 11, 23.9%)	Dissatisfied (*n* = 0, 0%)
6. Did the consultations and support group help you to deal more effectively with your problems?	Yes, it helped a lot (*n* = 32, 39.5%)	Yes, it helped some (*n* = 43, 53.1%)	No, it didn’t help (*n* = 6, 7.4%)	No, it was even worse afterwards (*n* = 0, 0%)
7. Overall, how satisfied are you with the workshops?	Yes, very satisfied (*n* = 43, 53.1%)	Yes, mostly satisfied (*n* = 29, 35.8%)	Indifferent or not very satisfied (*n* = 9, 11.1%)	Dissatisfied (*n* = 0, 0%)
8. If you needed help in the future, would you come back to our program?	Yes, without hesitation (*n* = 52, 64.2%)	Yes, I think so (*n* = 18, 22.2%)	I don’t know (*n* = 9, 11.1%)	No, never (*n* = 2, 2.5%)

### Children’s survey

Overall, global satisfaction was high. Of the workshop participants, 88% of the children gave a positive review (35.8% rating it as excellent and 52.2% as good) and 93.5% were pleased to have participated (45.7% were even very happy to have participated). An average rating was given by 13%, but none rated them as poor. A small number were not pleased to have participated (6.5%), but none were upset or dissatisfied to have partaken in the workshops.

The majority of children (82.5%) rated the workshops as helpful, however, 13% rated the workshop as only moderately helpful, and 4.3% as unhelpful. Three out of four children reported that the help received aided them to face their problems (76%), and no child found the workshops counterproductive (no child reported feeling worse afterwards). The majority of the children found that the workshops met their expectations (82.6%) either fully (23.9%) or partially (58.7%), and three fourths were satisfied with the help received (76.1%). Approximately one-fourth of participants felt indifferent towards the help received (23.9%), but none were dissatisfied.

Over half of the children claimed they would strongly recommend the workshop to a friend (54.3%) and a further third would probably recommend it (34.8%). One in ten would probably not recommend the workshop (10.9%), however none stated that they would strongly advise against it. If they felt the need, over half (58.7%) of the children would repeat the workshop, one third were unsure (37.7%), and a small percentage did not wish to participate again (4.3%).

Fifteen children wrote free-form commentaries. Four main themes emerged from the children’s feedback: need for extended follow-up (*n* = 3), normalizing grief (*n* = 2), difficulties speaking-up (*n* = 4), and praise for the team (*n* = 5). Two children were pleased to meet other bereaved children and to realize that they were “not the only child living with grief”. However, three children felt “uneasy” or “intimidated” by the group dynamic. Overall, the children were grateful towards the staff that were “kind”, “attentive listeners”, and “helpful”. Three children commented that four sessions was too short. An overview of the qualitative results is described in Table 3.

**Table 3:** Children’s free-form responses

**Theme**	**Quotes**
Need for extended follow-up (*n* = 3)	“Not enough sessions”
	“Maybe more sessions and closer to home…I found that 4 sessions was too short.”
	“Four sesions is too short and I would have liked to have done some painting.”
Normalizing grief (*n* = 2)	“I enjoyed participating in the workshop because it helped me to meet other kids that are in the same situation as me. I have a good memory (of the workshop) and I remember that I liked the fact that our parents didn’t see our drawings.”
	“It helped me to realize that I was not the only kid going through bereavement. I liked the hands-on activities that really helped me to reflect.”
Difficulties speaking-up (*n* = 4)	“I didn’t feel ready to talk at the time because I was too young.”
	“I was suffering…you didn’t hear me…I’m not saying it was because of you…I’m still suffering 5 years later.”
	“I was very intimidated”
	“The ambiance was difficult. I thought we would do more creative activities (painting). I felt uneasy when we had to take turns talking in front of everyone.”
Praise for the team (*n* = 5)	“Thank you for your support!”
	“Thank you for all the help you gave me, you helped free me from some of my fears and I hope the other children can say the same. I would like to redo the workshop with my brothers and sisters who didn’t do it last time if that’s possible.”
	“The sessions helped me a lot. I thank the staff who were attentive listeners.”
	“It was super cool and I would recommend it to other kids and teens.”
	“I loved the workshop and the staff were kind.”

### Parents’ survey

Overall satisfaction rates of the parents were equally high. Nearly all parents (92.8%) rated the quality of the workshop as good (37%) to excellent (55.6%), and most (88.9%) were satisfied to have participated in the workshop. A small percentage (7.4%) rated the quality as average or poor, and 11.1% were indifferent or less satisfied to have participated. None were dissatisfied.

Three-quarters of parents (76.6%) felt that their needs were either mostly or almost entirely met. Only 21% felt that few needs were met, and a small percentage (2.5%) felt that none of their needs were met by the workshop. Most parents (92.6%) felt that the consultations during the workshop and the parental support group sessions helped them to deal more effectively with their problems. Few felt that it did not help (7.4%) but none found them counterproductive. Over 90 % (91.3%) felt the workshop had met their expectations either partially (43.2%) or entirely (48.1%), and over 80 % (86.4%) were satisfied with the help received. However, nearly one-fourth (23.9%) were indifferent or less satisfied with the help received and 8.6% felt the workshop did not meet their expectations.

The overwhelming majority (93.9%) of parents would recommend the workshop to a friend and three quarters (74.1%) would strongly recommend it. Most parents would participate again in the workshop if they felt the need (86.4%), one in ten remain unsure if they would repeat their experience (11.1%), and a few would abstain from partaking in the workshop again (2.5%).

Forty-one parents wrote free-form commentaries. Five main themes emerge from the parents’ feedback: reducing isolation (*n* = 11), expressing and normalizing grief (*n* = 8), praise for the team (*n* = 8), need for extended follow-up (*n* = 11), and other improvements needed (*n* = 4). Eleven parents found a valuable support system in meeting other bereaved families. It allowed them to “feel less alone” in meeting other families “in the same situation”, “to emerge from [their] isolation”, and to realize that they “are not alone”. Some families even “forged strong friendships” and “kept in touch” long after the workshop was over. For eight families, the discussion groups were valuable help to “lift the taboo” on grief, to “put words to their feelings”, and to simply listen to other families’ stories like their own. The families were grateful towards the team for their “attentive listening”, “devotion”, “welcome”, and “accompaniment”. However, some aspects needed improvement: respecting age groups (avoid combining young children with teenagers), meeting hours compatible with school schedules, and post-intervention orientation and follow-up. Many families felt that four sessions were too few. One family suggested bi-annual follow-up meetings in order to “keep in touch”, and three families explicitly stated the need for extended in-depth care. An overview of the qualitative results is available in Table 4.

**Table 4:** Parents’ free-form responses

**Theme**	**Quotes**
Reducing isolation (*n* = 11)	“It helped us to move forward, to emerge from our isolation and see how others were pulling through.”
	“Thanks to the workshop we met other bereaved families with whom we forged a strong friendship.”
	“Meeting other families helped us to feel less alone and for that it’s a good initiative. Even 10 years later I’ve kept contact with a bereaved family, we send messages on the anniversary of our children’s death because for a parent there is nothing worse than being forgotten.”
	“Being in a group helped him to realise that he wasn’t alone; the other children as well.”
	“To realize that we weren’t the only family that had to go through this fatal tsunami. Today the wound is still there but the scar is softer.”
	“This group helped us keep our head above water. The peer-relationships forged have endured over time.”
	“A huge help. Even if you know it, you realise that you are not alone in this situation… we are all confronted with the same difficulties.”
Expressing and normalizing grief (*n* = 8)	“The workshop allowed me to pour myself out during the sessions.”
	“The worskhop is essential for finding the courage to speak, to just be able to listen to others’ experiences, and to be reassured on our [surviving] children’s health.”
	“Listening and discussing at that time of our lives with parents like ourselves who lost a child and learned to continue life differently helped us to express our feelings and listen to what we couldn’t yet verbalise.”
	“It wasn’t the activities that helped her the most, but rather the ability to talk to children who had lived the same thing.”
	“It helped us to lift the tabou and for the first time we felt understood.”
Praise for the team (*n* = 8)	“It was a rejuvenating place.”
	“The volunteers welcomed us, supervised us and helped us.”
	“Your skills were precious to us.”
Need for extended follow-up (*n* = 11)	“The sessions were a bit short and not everyone was able to share…we would have liked the meetings to have go one longer”
	“The length is perhaps too short. We only had four sessions but the workshop bore fruit.”
	“We would have liked more sessions: six? Or eight? The rhythm suited us though.”
	“The four sessions allowed my daughter to loosen up and speak, and she would have needed more extensive follow-up. Unfortunately we had to seek help elsewhere outside of the workshop.”
	“Organise follow-up meetings twice a year after the workshop to stay connected with the other children and parents.”
Other improvements needed (*n* = 4)	“For the workshop the blending of different ages is perhaps a drawback for an adolescent that is with small children and vice versa.”
	“The workshop hours were not compatible with school schedules.”
	“Our daughter was the only in her group to have lost her father in a car accident. The other children had lost their parents to illness, it wasn’t easy for her.”

## Discussion

We conducted an online email survey of 46 children and 81 parents that participated in the “Let’s Talk About It” bereavement workshop from 2011 to 2019. The families reported an overall high level of satisfaction regarding the intervention that was rated as good to excellent in quality. A majority of respondents considered their participation in the workshop helpful and in accordance with their expectations. Most would recommend the workshop to their friends and would repeat their experience if the need arose. The workshop helped them reduce isolation, express and normalize grief, and participate in creating a community among bereaved families.

The “Let’s Talk About It” workshop comprised of a series of four sessions lasting 90 min each over a period of 4 months. Group interventions are the most common form of bereavement service provided to children [27]. In comparison to other group interventions described in the literature, our workshop had fewer sessions and took place over a longer period of time. Most interventions comprise 8-10 sessions over a short timeframe of 2-3 months [27]. We developed a shorter four-session format in order to avoid leaving families with the impression that bereavement is a disease needing extensive professional help, but long enough to create a group dynamic amongst the children. Initial post-workshop verbal feedback from the families was positive in regards to its length. However, many families commented later via the survey that they would have preferred a longer workshop with more sessions. In regards to assessment, about 30% of published bereavement interventions for siblings provided pre- and post-bereavement care [27]. Children were assessed by a pediatrician before and after having participated in the workshop. If individual psychotherapy was indicated, the families were referred onwards. The families also had the option to repeat the workshop at a later time if desired. Although many people in our study commented that there were not enough sessions, only 37% of children said they would repeat the workshop “without hesitation”. Griese et al. provide an ongoing discussion group following their 10-week intervention, which families may partake in for however long they wish [32, 33]. Davies et al. provide an open discussion group for the families of their hospice with no restriction on participation duration, and the average length of attendance is 14 months [34]. In our study, a further 59.4% of children said they would probably repeat the workshop or were unsure, although the vast majority gave very positive ratings for the quality of the workshop and the help provided. This could possibly indicate that other forms of ongoing grief support are needed in addition to the workshop. Other forms of bereavement intervention described in literature are predominantly camps (typically one weekend to one week) and individual therapy [27]. Developing a short weekend or evening follow-up workshop could be one way to offer continual bereavement support. Our satisfaction survey thus highlights the potential need for family bereavement support over an extended period of time, including the provision of a variety of services.

It is important to take into account the whole family unit and the different needs of the parents and the child. In our workshop, the children attended the small group sessions and a parallel volunteer-led support group was available for parents. More parents responded that their needs were met (91.3% of parents versus 82.6% of children), and that their expectations were entirely met (48.1% of parents versus 23.9% of children). Some bereavement interventions performed qualitative assessment of their services [27, 35]. For the participating children, expressing and normalizing grief in a secure environment, reducing isolation and increasing social integration, or even simply having fun were of utmost importance [27]. Our results corroborate these findings. For the parents, the support was valuable on several levels- understanding their child’s grief, coping with their own grief, and receiving community peer support [27, 35]. Creating community among families and facilitating dialogue and mutual encouragement emerge as essential needs in bereavement care [27, 36, 37]. In addition, meeting families’ needs is an important component of professional satisfaction for the staff providing bereavement care [27].

With regards to the staff, all those who led the sessions were trained healthcare professionals (either psychologists or child psychiatrists) with specific training in childhood bereavement. The staff also benefited from frequent debriefing sessions. In the literature, staff qualification and training in pediatric grief support is varied: mental health professionals may comprise only half of bereavement service staff, not all may receive specific training and few seem to have access to debriefing sessions [27]. The pediatric palliative care team, the child psychiatry team and a volunteer organization collaborated in the creation of the workshop that was designed to be multidisciplinary. In our experience, having the sessions led by trained mental health professionals with a pediatric pre- and post-intervention assessment helps to screen for complicated grief and to refer children for individualized care when needed. Following this study, an additional psychologist was added to the team to accompany the pediatrician’s assessments and systematically contact families for follow-up approximately 4 months after the intervention.

On the other hand, some families were less enthusiastic about their workshop experience. Six and a half percent of children and 11.1% of adults were indifferent or less satisfied with their participation. Approximately one-fourth (23.9%) of the children and parents felt indifferent towards the help received, and some children rated the workshops as only moderately helpful (13%) or unhelpful (4.3%). A small percentage of parents (7.4%) equally rated the quality as average or poor and 8.6% of parents felt the workshop did not meet their expectations.

The greatest drawback mentioned by the children was their difficulty speaking-up within a group. During discussion, a ball is passed around from person to person indicating their turn to talk. If a child does not wish to speak, they can simply pass the ball on to their neighbor. We aim to allow them the freedom to say as much or as little as they feel comfortable. We assert that even simply listening to peers can be beneficial for the grief journey. However, it is assumed that some children unable to express themselves within this setting may be left feeling frustrated or ill at ease. This could be further influenced by the blending of ages in a group. Although our clinical experience with mixed age groups has been largely positive, perhaps not all needs are being met with large age gaps. We have found that the direct and open manner of the younger children disinhibits the older children, and the older children are protective and encouraging of the younger ones. This particular group dynamic is especially true of our bereaved sibling groups where the large age span is representative of a typical family dynamic with children of varying ages. However this environment could be intimidating for some, especially for the youngest ones. Following this study, we aspire to create workshops geared towards ages six and under to help address age-specific needs. In addition to different ages is the blending of different causes of death. Those who lost their loved one to chronic illness may have different bereavement needs to those who’s loved one died from an accidental or unforeseen cause.

Lastly, not all children may be ready to share their story in a group setting. Despite an intake screening process, the need for individual professional help may emerge later during, and perhaps thanks to, the workshop. Onward referral is always offered to these families and our post-workshop evaluation aims to ensure that each family’s needs and expectations were met. For an active and healthy group dynamic in a workshop, it is essential to verify at intake that the desire to participate is not simply that of the parents but also of the children.

Our study had some limitations. Our workshop was open to children who are bereaved of either a sibling or a parent. Although during the workshop children are separated into different groups depending on their relationship to the deceased, we did not differentiate respondents on this parameter in our survey. We acknowledge the fact that the needs and expectations of families bereaved of a child can differ to those bereaved of a parent. The results should thus be interpreted with caution. Furthermore, a short satisfaction survey is a simple method allowing a wide distribution and a high response rate. As a result, closed-ended questions and only one optional open-ended question restricted the depth and quality of the data collected. Within the workshop format, qualitative studies exploring family-specific needs and methods to better assist them over the long term are warranted. For example, our program is only comprised of four sessions and is perhaps too short to allow for this sense of community to develop in the long-term among children. Lastly, the time lapse between completion of the workshop and responding to the survey varied greatly—from a few months to 8 years. The fluctuating post-intervention interval represents an important recall bias. Nearly one in four children (23.9%) were indifferent to the help received, but the influence of time on bereavement trajectory could not be estimated in our study. Further quantitative studies evaluating program efficacy are merited.

## Conclusion

Childhood bereavement is commonplace, and its impact profound. Timely, developmentally appropriate and well-adapted intervention is paramount when caring for families. Few French pediatric bereavement interventions have been described and evaluated in literature. Our four-session workshop, led by trained grief counselors, aims to help reduce social isolation and foster coping skills through artistic creation and group discussion. Participants were largely satisfied with this form of bereavement intervention, but more extensive follow-up is desired. Encouraging community and mutual support among grieving families is fundamental in bereavement care. Our workshop model is feasible and acceptable for families, and further studies examining its efficacy are warranted.

## Data Availability

The datasets generated and analysed during the current study are available from the corresponding author on reasonable request.

## References

[1] Fauth B, Thompson M, Penny A. Associations between childhood bereavement and children’s background, experiences and outcomes. In: Secondary analysis of the 2004 mental health of children and young people in Great Britain data. London: National Children’s Bureau; 2009.

[2] Schonfeld DJ, Demaria T, Committee on Psychosocial Aspects of Child and Family Health, Disaster Preparedness Advisory Council (2016). Supporting the grieving child and family. Pediatrics.

[3] Eilegård A, Steineck G, Nyberg T, Kreicbergs U (2013). Psychological health in siblings who lost a brother or sister to cancer 2 to 9 years earlier. Psychooncology.

[4] Bylund-Grenklo T, Fürst CJ, Nyberg T, Steineck G, Kreicbergs U (2016). Unresolved grief and its consequences. A nationwide follow-up of teenage loss of a parent to cancer 6-9 years earlier. Support Care Cancer Off J Multinatl Assoc Support Care Cancer.

[5] Revet A, Bui E, Benvegnu G, Suc A, Mesquida L, Raynaud J-P (2020). Bereavement and reactions of grief among children and adolescents: present data and perspectives. L’Encéphale.

[6] Bolton JM, Au W, Chateau D, Walld R, Leslie WD, Enns J (2016). Bereavement after sibling death: a population-based longitudinal case-control study. World Psychiatry Off J World Psychiatr Assoc WPA.

[7] Rosenberg AR, Postier A, Osenga K, Kreicbergs U, Neville B, Dussel V (2015). Long-term psychosocial outcomes among bereaved siblings of children with cancer. J Pain Symptom Manag.

[8] Björkenstam E, Burström B, Vinnerljung B, Kosidou K (2016). Childhood adversity and psychiatric disorder in young adulthood: an analysis of 107,704 Swedes. J Psychiatr Res.

[9] Romano H (2017). Le deuil chez l’enfant: spécificités selon les âges. Neuropsychiatr Enfance Adolesc.

[10] Stikkelbroek Y, Bodden DHM, Reitz E, Vollebergh WAM, van Baar AL (2016). Mental health of adolescents before and after the death of a parent or sibling. Eur Child Adolesc Psychiatry.

[11] Mack KY (2001). Childhood family disruptions and adult well-being: the differential effects of divorce and parental death. Death Stud.

[12] Fletcher J, Vidal-Fernandez M, Wolfe B (2018). Dynamic and heterogeneous effects of sibling death on children’s outcomes. Proc Natl Acad Sci U S A.

[13] Burrell LV, Mehlum L, Qin P (2021). Co-occurrence of psychosocial sequelae in bereaved offspring. J Affect Disord.

[14] Berg L, Rostila M, Saarela J, Hjern A (2014). Parental death during childhood and subsequent school performance. Pediatrics.

[15] von Sydow K, Lieb R, Pfister H, Höfler M, Wittchen H-U (2002). What predicts incident use of cannabis and progression to abuse and dependence?. Drug Alcohol Depend.

[16] Giordano GN, Ohlsson H, Kendler KS, Sundquist K, Sundquist J (2014). Unexpected adverse childhood experiences and subsequent drug use disorder: a Swedish population study (1995-2011): childhood trauma, drug use disorder. Addiction.

[17] Ortin-Peralta A, Keski-Säntti M, Gissler M, Veijola J, Sourander A, Duarte CS. Parental suicide attempts and offspring's risk of attempting or dying by suicide: does the timing of a parental suicide attempt matter? Psychol Med. 2021;18:1-10. 10.1017/S0033291721002397. Epub ahead of print. 10.1017/S0033291721002397 34140058

[18] Eilertsen M-EB, Eilegård A, Steineck G, Nyberg T, Kreicbergs U (2013). Impact of social support on bereaved siblings’ anxiety: a nationwide follow-up. J Pediatr Oncol Nurs Off J Assoc Pediatr Oncol Nurses.

[19] Lövgren M, Jalmsell L, Eilegård Wallin A, Steineck G, Kreicbergs U (2016). Siblings’ experiences of their brother’s or sister’s cancer death: a nationwide follow-up 2-9 years later. Psychooncology.

[20] Sveen J, Eilegård A, Steineck G, Kreicbergs U (2014). They still grieve-a nationwide follow-up of young adults 2-9 years after losing a sibling to cancer. Psychooncology.

[21] Worden JW, Silverman PR (1996). Parental death and the adjustment of school-age children. OMEGA J Death Dying.

[22] Greenwald N, Barrera M, Neville A, Hancock K (2017). Feasibility of group intervention for bereaved siblings after pediatric cancer death. J Psychosoc Oncol.

[23] Stein A, Dalton L, Rapa E, Bluebond-Langner M, Hanington L, Stein KF (2019). Communication with children and adolescents about the diagnosis of their own life-threatening condition. Lancet Lond Engl.

[24] Hanus M (2007). Les deuils dans la vie: deuils et séparations chez l’adulte et chez l’enfant.

[25] Shear MK, Simon N, Wall M, Zisook S, Neimeyer R, Duan N (2011). Complicated grief and related bereavement issues for DSM-5. Depress Anxiety.

[26] Chapter 5, Bereavement during childhood and adolescence. In: Osterweis M, Solomon F, Green M, editors. Bereavement: reactions, consequences, and care. Washington, D.C.: National Academies Press; 1984. Available from: http://www.nap.edu/catalog/8. Cited 2021 Feb 14. 25032459

[27] Ridley A, Frache S. Bereavement care interventions for children under the age of 18 following the death of a sibling: a systematic review. Palliat Med. 2020. 10.1177/0269216320947951. 10.1177/0269216320947951 32807009

[28] Suc A, Blandin I, Lutgen V, Bayle M, Cayzac D, Raynaud J-P (2013). Enfants endeuillés: expérience des ateliers médiatisés «Histoire d’en parler». Médecine Palliat Soins Support - Accompagnement - Éthique.

[29] Eysenbach G (2004). Improving the quality of Web surveys: the Checklist for Reporting Results of Internet E-Surveys (CHERRIES). J Med Internet Res.

[30] Thomas J, Harden A (2008). Methods for the thematic synthesis of qualitative research in systematic reviews. BMC Med Res Methodol.

[31] Packman W, Horsley H, Davies B, Kramer R (2006). Sibling bereavement and continuing bonds. Death Stud.

[32] Griese B, Burns M, Farro SA (2018). Pathfinders: promoting healthy adjustment in bereaved children and families. Death Stud.

[33] Griese B, Burns MR, Farro SA, Silvern L, Talmi A (2017). Comprehensive grief care for children and families: policy and practice implications. Am J Orthop.

[34] Davies B, Collins J, Steele R, Cook K, Distler V, Brenner A (2007). Parents’ and children’s perspectives of a children’s hospice bereavement program. J Palliat Care.

[35] Bergman A-S, Axberg U, Hanson E (2017). When a parent dies - a systematic review of the effects of support programs for parentally bereaved children and their caregivers. BMC Palliat Care.

[36] Laing CM, Moules NJ (2015). Children’s cancer camps: a way to understand grief differently. Omega.

[37] Laing CM, Moules NJ (2016). ‘It’s not just camp!’: understanding the meaning of children’s cancer camps for children and families. J Pediatr Oncol Nurs Off J Assoc Pediatr Oncol Nurses.

